# Relative expression analysis of light‐harvesting genes in the freshwater alga *Lympha mucosa* (Batrachospermales, Rhodophyta)

**DOI:** 10.1111/jpy.12967

**Published:** 2020-02-10

**Authors:** Joshua R. Evans, Morgan L. Vis

**Affiliations:** ^1^ Department of Environmental and Plant Biology Ohio University Athens Ohio 45701 USA

**Keywords:** ecophysiology, gene expression, Nemaliophycidae, Photoacclimation, photosynthesis, qPCR, temperate streams

## Abstract

Members of the freshwater red algal order Batrachospermales are often described as shade‐adapted. Nevertheless, recent ecophysiological studies have demonstrated species‐level differences in acclimation to a range of irradiances. *Lympha mucosa* occurs in open and shaded portions of temperate streams and is abundant during summer months, suggesting it tolerates high and low irradiances. Specimens of *L. mucosa* were collected from open (sun‐acclimated) or shaded (shade‐acclimated) sites and exposed to low (<20 μmol photons · m^−2^ · s^−1^) or high (220 μmol photon · m^−2^ · s^−1^) light for 72 h to examine mechanisms of photoacclimation at the transcriptional level. High‐throughput sequence data were used to design specific primers for genes involved with light harvesting and these were quantified with qPCR. The greatest significant difference in transcript abundances was observed in the *psa*A gene (Photosystem I P700 apoprotein), and site‐type had an effect on these responses. Shade‐acclimated thalli were 22‐fold down‐regulated at high light, whereas sun‐acclimated thalli were only 5‐fold down‐regulated. Another gene involved with Photosystem I (*pet*F ferredoxin) was down‐regulated at high light, but only individuals from the shaded site were significantly different (4‐fold). In thalli from both sites, *cpe*A (Phycoerythrin alpha chain) was down‐regulated at high light. Although not statistically significant, patterns consistent with previous physiological and transcriptomic studies were uncovered, namely the inverse response of transcriptional activity in genes that encode phycobiliproteins. In support of previous ecophysiological studies of freshwater red algae, these data indicate significant transcriptional changes involving Photosystem I and phycobiliprotein synthesis are required to tolerate and grow at various irradiances.

**Abbreviations**
*apc*A: allophycocyanin alpha chain gene
*cpc*A: phycocyanin alpha chain gene
*cpe*A: phycoerythrin alpha chain gene
*eRF*3: ethylene‐responsive transcription factor 3 gene
HL: high‐light treatment
*HV60*
: low molecular mass early light‐inducible protein HV60 gene
LL: low‐Light treatment
*pet*F: ferredoxin gene
*psa*A: photosystem I P700 apoprotein gene
*psb*A: photosystem II protein D1 gene
PS: photosystem
*rps*3: ribosomal protein subunit 3 gene
SA: sun‐acclimated
SH: shade‐acclimated

Red algae like other oxygenic autotrophs have two photosystem (PS) reaction centers that serve as the sites of light harvesting and photophosphorylation (Lepetit and Dietzel 2015). Although most photosynthetic eukaryotes contain other chlorophylls that comprise the light‐harvesting antenna of PS II, red algae utilize light‐harvesting phycobiliprotein complexes known as phycobilisomes (Gantt 1990). The differences in the absorption spectra of these unique phycobilins relative to chlorophylls allow red algae to take advantage of energy from wavelengths poorly absorbed by most green algae and plants (Gantt 1990). Other proteins associated with light harvesting and photoprotection in red algae include high‐light‐induced proteins, one‐helix proteins, two‐helix stress‐enhanced proteins, and chl *a/b* binding‐like proteins (RedCAP; Engelken et al. 2010).

The ~5% of red algae that occur in freshwater environments has a worldwide distribution, particularly in streams with low organic pollution and little canopy modification (Sheath 1984). The strictly freshwater order Batrachospermales is widespread, but most taxa inhabit first‐ to third‐order streams (Entwisle et al. 2009, Sheath and Vis 2015). The distribution and biogeographic patterns in the Batrachospermales are diverse, but there are limited physiological and genetic data for factors that may affect distributions, phenology, and dispersal (Vis 2016).

Light regime is an important environmental factor that controls the growth, distribution, and seasonality of freshwater red algae (Sheath 1984, Necchi et al. 1999). In temperate regions, many streams have seasonal changes in light regimes related to deciduous tree canopy cover that affect red algal distribution and seasonality. For example, Drerup and Vis (2014) noted differences in the phenology of *Batrachospermum gelatinosum* from geographically close temperate streams with the gametophyte thalli being spring ephemerals in the smaller stream and perennial in the larger stream. The spring ephemeral seasonality was likely due to greater canopy cover and consequently a lack of sufficient light for basic photosynthetic requirements.

Previous ecological investigations of light regime on growth and photosynthetic rates in freshwater red algae have concluded that the Batrachospermales are primarily shade‐adapted. These investigations included transplant experiments of two *Batrachospermum* spp. from shaded sites that disintegrated in the first few days of exposure to open sites (Parker et al. 1973); and ecophysiological studies of photosynthetic rates in several batrachospermalean taxa that become light saturated at irradiances <250 μmol photons · m^−2^ · s^−1^ (e.g., Leukart and Hanelt 1995, Necchi and Zucchi 2001, Necchi 2005, Drerup et al. 2015). However, there are several species that occur naturally in consistently higher light intensities, some as high as 2,400 μmol photons · m^−2^ · s^−1^ (Bautista and Necchi 2007), and different taxa occurring in the same stream can vary in their response to changes in light intensities (Drerup et al. 2015). Furthermore, some shade‐adapted species may actually tolerate a much wider range of irradiance than previously believed. *Batrachospermum turfosum* displays photosynthetic characteristics of shade‐adapted algae (low‐light compensation and saturation points), yet this species has been shown to tolerate light intensities up to ~1,000 μmol photons · m^−2^ · s^−1^ (Aigner et al. 2017). Hence, it is probable that red algal photosystems have evolved adaptations for a wide range of light.

The term “acclimation” refers to the ability of an organism to respond to environmental changes within the limits of its genome, whereas “adaptation” refers to a response formed through alterations to the genome over generations (Gantt 1990). Studies have shown various types of photoacclimation utilized by freshwater red algae including differential adjustments to pigment content and distinct photosynthetic characteristics (e.g., low saturation and compensation points), which have been documented in culture and field studies of eight freshwater red algal taxa (Kaczmarczyk and Sheath 1991, Bautista and Necchi 2007, Drerup et al. 2015). Potential adjustments for photoacclimation in red algae include changes in the size or number of photosystems and/or phycobilisomes on the thylakoid, and changes in photopigment content, specifically the ratio of phycocyanin and phycoerythrin (Gantt 1990, Kaczmarczyk and Sheath 1991, Bautista and Necchi 2007).

The majority of photosynthetic studies of freshwater red algae have been at the whole‐thallus level, but understanding photoacclimation strategies and other photosynthetic processes likely involves determining the changes that occur at both the individual and molecular levels (Talarico and Maranzana 2000). In a recent transcriptome‐wide survey of the batrachospermalean *Sheathia arcuata,* Nan et al. (2018) showed that several transcripts involved with photosystem antenna and electron transfer were up‐regulated in low light. With support through evidence of increased transcriptional activity in red algal plastids (Minoda et al. 2005), transcriptional regulation is a potential mechanism for cellular adjustments related to photoacclimation in red algae.

The goal of the current study was to generate genetic data related to photoacclimation in a freshwater red alga that occurs in a wide range of irradiances. *Lympha mucosa* is a mucilaginous, filamentous taxon in the Batrachospermales that occurs abundantly throughout the summer months in both shaded and open sites of a single stream. Previously, Drerup et al. (2015) reported high photosynthetic efficiencies and saturation points for this taxon (as *Batrachospermum* sp.), but little is known of the adjustments at the molecular level. Therefore, a near‐complete plastid genome was utilized as a tool for specific primer design to target light‐harvesting genes in relative expression profiling experiments of light acclimation. It was hypothesized that photoacclimation in freshwater red algae involves, at least in part, active transcriptional regulation of genes encoding important light‐harvesting proteins. Therefore, genes encoding components of the red algal light‐harvesting machinery were expected to show differential expression after exposure to varying irradiances.

## Materials and methods

### Sample collection and experimental design

Samples of *Lympha mucosa* were collected from the Kinniconick Creek, KY (38.496667 N, 83.257222 W) in July 2016. Water conditions (temperature, pH, specific conductance) and light quantity were measured at midday. Specific conductance was measured with a Waterproof ECTestr low (Oakton, Vernon Hills, IL, USA), pH with a Waterproof pHTestr 30 Double Junction (Oakton) and temperature with a thermometer. Light quantity was measured using a Li‐250A light meter equipped with a Li‐192 underwater quantum sensor (Li‐Cor, Lincoln, NE, USA). Specific conductance was 110 μS · cm^−1^, pH was 6.6, and the water temperature was 29°C. Light intensity at the river midsection (open site) was 1132 μmol photons · m^−2^ · s^−1^ and the edge (shaded site) was 76 μmol photons · m^−2^ · s^−1^. Facing downstream, a cliff on the right side of the streambank shades the edge site throughout the day, resulting in consistently low‐light intensity during months of high canopy cover. In all, 12 samples were collected from the river midsection (i.e., open site, sun‐acclimated) and the river edge (shaded site, shade‐acclimated) for a total of 24 samples (Fig. S1 in the Supporting Information). These samples were kept in the dark on ice for transport and storage before placing in different light treatments. Additional samples were collected for DNA extraction.

Samples for DNA extraction were desiccated in silica gel. All other samples (~1 g wet weight each) were immediately transferred to 70 mL culture flasks (Corning Inc., Corning, NY, USA) with filtered stream water and placed on a Lab‐line orbital shaker (Lab‐line Instruments Inc., Dubuque, IA, USA) at a medium setting to simulate water flow. The shaker was located in a Conviron CMP6050 walk‐in growth chamber (Controlled Experiments Ltd., Winnipeg, Manitoba, Canada) equipped with 60‐W cool‐white fluorescent and/or 40‐W incandescent bulbs. Sun‐ and shade‐acclimated samples were split equally into two treatments of low (<20 μmol photons · m^−2^ · s^−1^) or high (220 μmol photons · m^−2^ · s^−1^) light. The high‐light intensity was chosen based on the results of photosynthesis–irradiance (P‐I) curves for individuals from the same population in Drerup et al. (2015). Like most of the other red algal species examined in that study, the onset of photoinhibition and reductions in photosynthetic rates occurred at irradiances >240 μmol photons · m^−2^ · s^−1^. The quantity of light in the growth chamber was set to high‐light conditions, and low‐light conditions were achieved by covering flasks with two layers of 50% shade cloth. Light conditions for each treatment were verified using the Li‐250A light meter. All other growth chamber conditions were kept constant: temperature 22°C, relative humidity 50–70%, and day length 16:8 h light:dark. Photoacclimation can take a few days to a few weeks in red algal cells (e.g., Gantt 1990) and it is difficult to culture batrachospermalean gametophytes longer than 1 week. Therefore, samples were grown in the growth chamber for 72 h to allow enough time to acclimate to new light conditions, but without visible thallus deterioration. Following the 72 h growth period, all samples were flash frozen in liquid nitrogen and stored in a freezer at −80°C until RNA extraction.

### DNA extraction and RNA extraction

Silica‐desiccated samples had DNA extracted with a NucleoSpin^®^ Plant II DNA kit (Macherey‐Nagel) following the manufacturer's protocol with a potassium acetate (KOAc) addition for extracting from polysaccharide‐rich algae (e.g., Saunders 1993, Dos Reis Falcão et al. 2008). Unpurified DNA was cleaned with a PowerClean^®^ Pro DNA Clean‐Up Kit (Mo Bio Laboratories, Carlsbad, CA, USA) and DNA purity was assessed with a NanoDrop Spectrophotometer (Thermo Scientific, Waltham, MA, USA).

Frozen Lympha *mucosa* samples for total RNA isolation were processed within 4 months of collection to reduce the potential for sample degradation. Total RNA was isolated using a NucleoSpin^®^ RNA Plant Kit (Macherey‐Nagel) following the manufacturer's protocol with KOAc addition as above, and DNA digestion. However, for RNA samples, the KOAc incubation period was performed at −20°C, followed by 4°C centrifugation at 12,000*g* for 10 min to reduce RNA degradation. RNA concentration and purity were assessed using a NanoDrop spectrophotometer, and an Agilent 2100 Bioanalyzer. RNA samples were stored in a freezer of −80°C.

### Plastid genome assembly

High‐throughput sequencing of the extracted genomic DNA was performed by the Ohio University Genomics Facility (OUGF) personnel on an Illumina MiSeq platform with Nextera™ sequencing primers and a MiSeq Reagent Kit v3 (600 cycle; Illumina Inc., San Diego, CA, USA). The first sequencing run was overloaded with bacterial contamination; therefore, a second sequencing run was performed with an Illumina MiSeq Reagent Kit v2 (300 cycle). Raw reads were assessed for per base sequence quality, content, length distribution, and Kmer content with FastQC v0.11.5 (Andrews 2016). Reads of low quality (<Q20) or short length (<50 bp) were discarded. Filtered reads were de novo assembled either with CLC Genomics Workbench 10.0.1 (CLC Bio, Aarhus, Denmark) or with the SPAdes 3.10.0 (Bankevich et al. 2012) assembler plug‐in for Geneious 10.1.3 (Kearse et al. 2012) using default parameters. Contiguous sequences (contigs) were searched against a local BLAST database with the *Kumanoa americana* plastid genome used as a query (GenBank accession NC031178; Lee et al. 2016). Plastid contigs were stitched together and used as a reference for remapping in CLC with a similarity fraction of 0.85–0.9 and length fraction of 0.8–0.85, or in Geneious v.10.13 with medium sensitivity/fast, to verify the near‐complete sequence. Gene annotations and predictions were performed with Pfam 30.0 (Finn et al. 2016) and MFannot (Beck and Lang 2010), and annotations for rRNA and tRNA genes were detected using RNAmmer (Lagesen et al. 2007) and tRNAdb (Chan and Lowe 2009). The near complete Lympha *mucosa* plastid sequence was deposited in GenBank (MN509464) and raw high‐throughput sequence data are available in the SRA database (PRJNA574195).

### RT‐qPCR primer design

Seven genes were targeted for RT‐qPCR. From the Lympha *mucosa* plastid assembly, sequence data were used for primer design of six selected protein‐coding genes; these genes were chosen based on their function in light harvesting and/or use in previous studies (Ritz et al. 2000, Engelken et al. 2010, Lepetit and Dietzel 2015). The target genes encode subunits of both photosystem reaction centers (*psa*A, Photosystem I P700 chlorophyll a apoprotein; *psb*A, Photosystem II protein D1), and a gene encoding a photophosphorylated electron acceptor (*pet*F, PetF ferredoxin I). For the light‐harvesting phycobilisome protein complex, subunits encoding each of the primary pigment binding proteins were chosen (*apcA*, Allophycocyanin alpha chain; *cpc*A, Phycocyanin alpha chain; *cpe*A, Phycoerythrin alpha chain). For the final gene, a fragment (534 bp) of the nuclear‐encoded *HV*60 (low molecular mass early light‐inducible protein HV60) was mined from the high‐throughput sequencing data using BLASTx searches, with 65% similarity to putative *HV60* CDS sequences from *Griffthsia japonica* and *Porphyra umbilicalis*. The *L. mucosa HV60* DNA sequence was deposited in GenBank (MN411325).

Two endogenous controls, one each from the nuclear and plastid genome compartments, were chosen for data normalization. The nuclear‐encoded *eRF*3 (Ethylene‐responsive transcription factor 3) gene was selected from a comparative study of appropriate endogenous controls for red algal studies (Kowalczyk et al. 2014), and the plastid‐encoded *rps*3 (ribosomal protein subunit 3) was chosen from a comparison of plastid endogenous controls in vascular plants (Cortleven et al. 2009). These genes have been shown to be stably expressed in at least 15 different environmental conditions, including changes in light regime, and genes from each genomic compartment were chosen for more accurate reflections of transcriptional activity of each target (Cortleven et al. 2009, Kowalczyk et al. 2014). An analysis of expression stability was conducted for all quantified genes using the geNorm algorithm (Vandesompele et al. 2002) in qbase + 3.2 (Biogazelle, Zwijnaarde, Belgium – www.qbaseplus.com). The Lympha *mucosa eRF*3 DNA sequence was deposited in GenBank (MN509465).

Primer sets for each gene were designed using NCBI Primer‐BLAST (Ye et al. 2012; Table 1). Specifications were set with an optimal melting temperature (Tm) of 60°C, maximum Tm difference of 1°C between primer pairs, and GC content of 40–60%. The specificity of these synthetic oligonucleotides was tested in silico on the Lympha *mucosa* plastid genome and through a local BLASTn search on the contig database. Primer sets were tested for amplification using gDNA (as there were no intronic regions present in these genes). These products were purified using an UltraClean™ PCR Clean‐up DNA purification kit (Mo Bio, Carlsbad, CA, USA) and Sanger sequenced at the OUGF. Successful primer sets were used for RT‐qPCR.

**Table 1:** Primer sets used for RT‐qPCR and amplicon lengths for each gene

Gene (amplicon length)	Primer	Sequence (5′–3′)	Expected *T*m (°C)	GC content (%)
*apc*A (170 bp)	apcAF1	ATGACTGCAACGTGTTTGCG	60.32	50.0
	apcAR1	GCACGTAAGCCTTCTGCAAC	60.11	55.0
*cpc*A (197 bp)	cpcAF1	TAACAAGCAGCTCGCAACGA	60.60	50.0
	cpcAR1	CTCATCCATGGGTCCAGTTGAA	60.03	50.0
*cpe*A (152 bp)	cpeAF1	TGGCACAGGACCTTTAGACG	59.68	55.0
	cpeAR1	CTACACCTGCTTGAGCAGACA	60.00	52.4
*HV60* (nuclear) (169 bp)	LCAPF1	ATGTCATGTTCGGCTGGCTT	60.32	50.0
	LCAPR1	AAGTGGATGTGAGCGACGAG	60.11	55.0
*pet*F (106 bp)	petFF1	TTGGATGCAGCGGAAGATCAA	60.34	47.6
	petFR1	CAGACTGGTCAACCGAACCTT	60.20	52.4
*psa*A (171 bp)	psaAF1	GGGCACATTTTGTATGGGCTTT	60.03	45.5
	psaAR1	AATGTGCAACTCCGACTGCT	60.25	50.0
*psb*A (143 bp)	psbAF1	AGTCAAGGGCGCGTAATCAA	60.04	50.0
	psbAR1	GGTGCAACTAAAGCAACTGGG	60.00	52.4
*eRF3* (nuclear ref.) (159 bp)	eRF3F1	TGAAAGTCAGGGCAACGGAA	59.82	50.0
	eRF3R1	GGGCACAATGGTAAAGGGGA	59.96	55.0
*rps*3 (plastid ref.) (150 bp)	rps3F1	GCCAATAGACGTGCTTCTGTG	59.61	52.4
	rps3R1	AACAGCTAGACCAGGAATTGTCC	60.31	47.8

### qRT‐PCR of light‐harvesting genes

A total of three biological replicates were used from each treatment. RNA samples were synthesized into cDNA using the iScript™ cDNA Synthesis Kit (Bio‐Rad, Hercules, CA, USA) following the manufacturer's protocol. For RT‐qPCR, PowerUp™ SYBR™ Green Master Mix (Thermo Fisher Scientific, Waltham, MA, USA) was used with ROX as a passive reference dye. Amplification was performed on either an Agilent Stratagene Mx3000P qPCR machine (Agilent Technologies, Santa Clara, CA, USA) or a Bio‐Rad CFX96 Touch™ Real‐Time PCR Detection System (Bio‐Rad Laboratories, Hercules, CA, USA). Template concentration and primer efficiencies were optimized by performing a 5‐fold dilution series to generate a standard curve, and the quantification cycle (C_q_) threshold and range for each target was determined using the instrument software (Table S1 in the Supporting Information). Each reaction consisted of 10 μL SYBR master mix (with ROX), 1 μL of each 0.5–0.75 μM forward and reverse primer, 6 μL nuclease‐free dH_2_O, and 2 μL diluted cDNA. Standardization of genes was performed with technical duplicates and amplification specificity was assessed with a heat curve (55–95°C cycles following each amplification run). All successfully optimized genes were included for relative expression profiling using 10 μL SYBR master mix (with ROX), 1 μL of each 0.75 μM forward and reverse primer, 6 μL nuclease‐free dH_2_O, and 2 μL cDNA diluted 1:5 or 1:25 (depending on target gene). Other PCR conditions followed manufacturer's protocol. All samples were amplified with technical triplicates and each gene set had NT (no template) controls performed on the same plate. NRT (no reverse transcriptase) controls were performed on RNA samples using PCR amplification with parameters and primer concentrations listed above. The raw C_q_ data set can be accessed in Table S2 in the Supporting Information.

### Gene expression analysis

Raw C_q_ data for each target gene were normalized to the geometric mean of the two endogenous controls to calculate ΔC_q_ values for each sample replicate, and these data sets were tested for normality using a Shapiro–Wilks test in R (R Core Team 2018). Relative expression of each target gene was calculated as the fold change between treatments using the ΔΔC_t_ method on a log_2_ scale (Schmittgen and Livak 2008). Differences in expression between each pair of treatments and the effect of original site location were assessed for statistical significance using a two‐way analysis of variance (ANOVA) with a post hoc Tukey HSD completed in R (R Core Team 2018). Results from the statistical analysis are in Table S3 in the Supporting Information.

## Results

### Plastid genome

A total of 186,825 paired‐end reads were assembled into the ≥189,825 bp Lympha *mucosa* partial plastid genome with 146X average sequencing depth (Fig. S2 in the Supporting Information). The genome was AT‐rich (GC content 28.4%) and had 239 genes, of which seven were partial sequences. Overall, 198 protein‐coding genes were annotated and included seven open‐reading frames (ORFs) and 21 *ycf* genes. A double‐copy ribosomal operon in the *L. mucosa* plastid genome was present as an inverted repeat (IR) region consisting of the 5S, 16S and 23S rRNAs, and two tRNAs. Independent remapping of the intergenic space next to each IR and their respective gene neighbors resolved their placement in the *L. mucosa* assembly. In addition to the tRNAs in the ribosomal operons, 29 other tRNAs encoding all 20 amino acids were annotated throughout the genome. Finally, two highly conserved group II introns in the Nemaliophycidae were detected as partial sequences in a tRNA for methionine and in the *chlB* gene (Fig. S2).

### Gene expression analyses

Transcription for six of the seven plastid‐encoded targets and *HV60* was successfully quantified. The gene *apcA* was discarded from the analyses due to a failure to obtain ~100% primer efficiency during optimization of the PCR conditions. Optimization of all other target genes had PCR efficiencies of 80–107% and R^2^ of 0.993–0.999 (Table S1).

**Figure 1:**
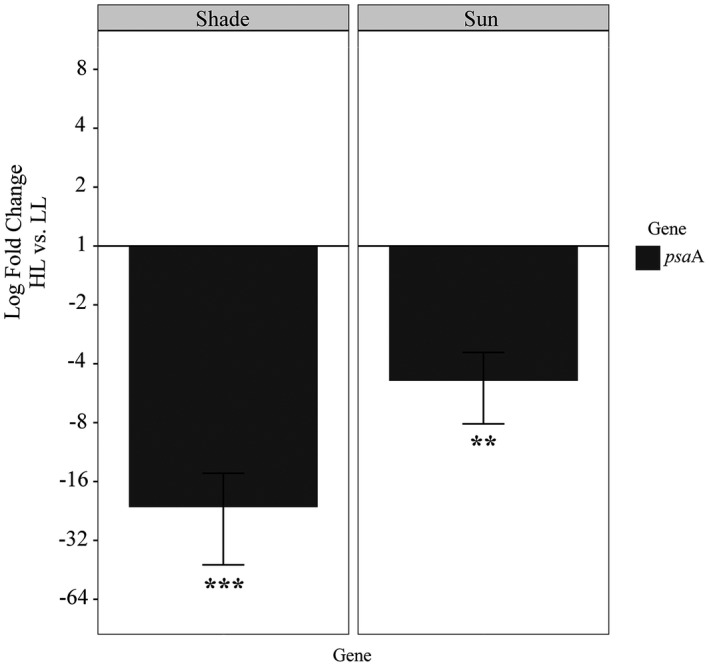
Log_2_ fold changes of *psa*A transcripts in shade‐ and sun‐acclimated *Lympha mucosa* thalli exposed to high light and low light. Data were normalized to the geometric mean of the two endogenous controls. Significant changes in relative transcript abundance between high‐ and low‐light treatments were determined using a two‐way ANOVA and are denoted by asterisks (**P <* *0.01, ***P <* *0.001). The magnitude of change observed between the two acclimation groups was significantly different based on a post hoc Tukey HSD test.

**Figure 2:**
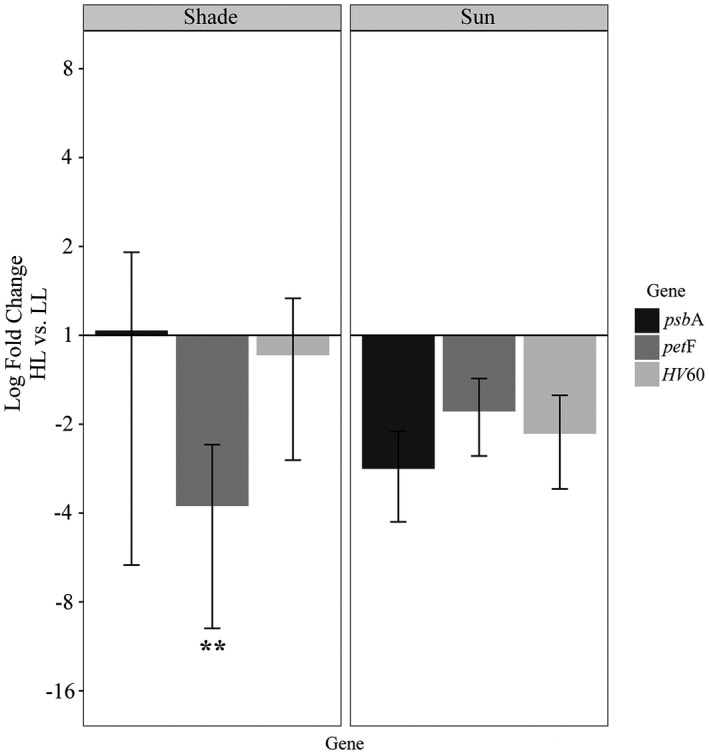
Log_2_ fold changes of *psb*A, *pet*F, and *HV* 60 transcripts in shade‐ and sun‐acclimated *Lympha mucosa* thalli exposed to high light and low light. Data were normalized to the geometric mean of the two endogenous controls. Significant changes in relative transcript abundance between high‐ and low‐light treatments were determined using a two‐way ANOVA and are denoted by asterisks (**P <* *0.01).

The target genes encoding subunits of PS I (*psa*A and *pet*F) were differentially expressed at low light and high light. For *psa*A, relative expression was differentially regulated (ANOVA *F*_1,8_ = 78.360, *P *<* *0.0005) and there was a significant effect caused by acclimation type; regulation of *psa*A in shade‐acclimated thalli was 22‐fold down‐regulated (Tukey HSD_8_ = −11.611, *P *<* *0.0001), while in sun‐acclimated thalli it was only down‐regulated 5‐fold (Tukey HSD_8_ = −6.092, *P *<* *0.01; Fig. 1). Transcript abundance of *pet*F was similarly affected in both acclimation types based on light treatment. At high light, *pet*F in shade‐acclimated thalli was significantly down‐regulated 4‐fold (ANOVA *F*_1,8_ = 22.445, *P *<* *0.01), but the apparent down‐regulation in sun‐acclimated thalli was not significant (Fig. 2). Other target genes involved with photosystem proteins (*HV*60, *psb*A) were not differentially expressed with statistical significance, and there were several outliers from shade‐acclimated samples in measurements for both targets. However, the 2‐fold down‐regulation of *HV*60 at high light in the sun‐acclimated group had a *P* value close to the 0.05 significance threshold (ANOVA *F*_1,8_ **= 4.909, *P *=* *0.06; Table S3).

**Figure 3:**
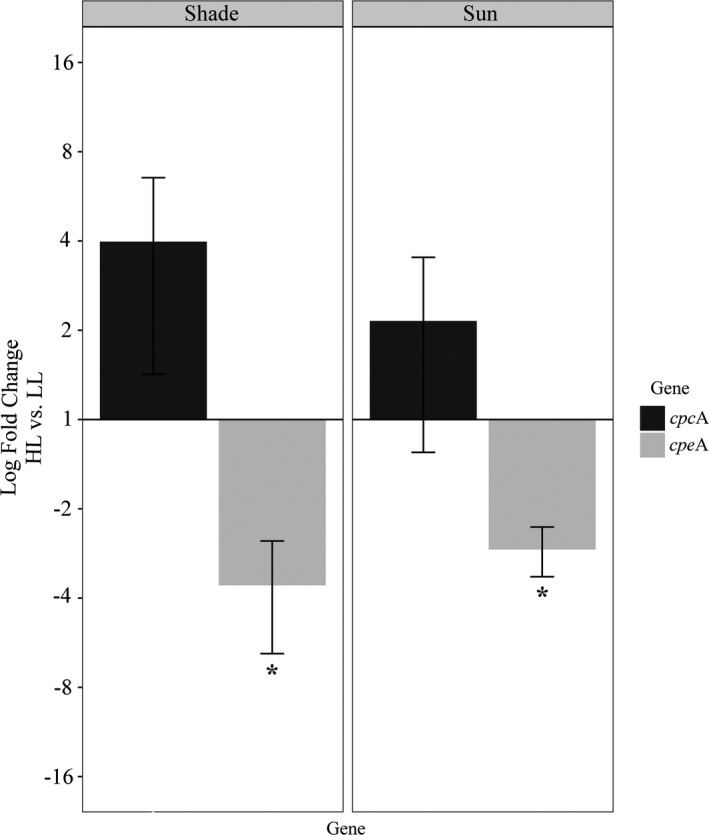
Log_2_ fold changes of *cpc*A and *cpe*A transcripts in shade‐ and sun‐acclimated *Lympha mucosa* thalli exposed to high light and low light. Data were normalized to the geometric mean of the two endogenous controls. Significant changes in relative transcript abundance between high‐ and low‐light treatments were determined using a two‐way ANOVA and are denoted by asterisks (*P <* *0.05).

The genes encoding phycobilisome proteins (*cpc*A, *cpe*A) showed an inverse transcriptional response between light treatments in both acclimation types. At high light, *cpe*A was significantly down‐regulated 3‐ and 4‐fold in sun‐ and shade‐acclimated samples, respectively (ANOVA *F*_1,8_ = 8.349, *P *<* *0.05; Fig. 3). Although *cpc*A did not have significant differential expression, the gene was 2‐ to 4‐fold up‐regulated at high light (Fig. 3).

## Discussion

### Differential expression of select light‐harvesting genes

High light prompted substantial differentiation in the relative expression of *psa*A for both sun‐ and shade‐acclimated thalli, but the 22‐fold down‐regulation in shade‐acclimated thalli was the greatest response observed in this study. Shade‐acclimated thalli were collected from a site in the stream receiving 76 μmol photons · m^−2^ · s^−1^, and those thalli that were exposed to lower light (<20 μmol photons · m^−2 ^·^ ^s^−1^) for 72 h had up‐regulated expression of *psa*A relative to the high‐light treatment. Significant down‐regulation in *psa*A at high light may be supported by a finding of decreased *chl. a* content for freshwater red algae cultures in similar conditions (Bautista and Necchi 2007), as chl*. a* is predominantly located in PS I (Gantt 1990).

Interestingly, PS I in red algae differs from other photosynthetic clades by having four protein subunits involved with oxygen evolution, but the overall role of these subunits is unknown (Grouneva et al. 2013). The differential expression of *psa*A between these treatment groups indicates significant adjustments occur to the number or size of PS I units on the thylakoid surface at different light intensities. These adjustments allow Lympha *mucosa* to harvest sufficient light energy from low‐light environments present in the shaded sites but also to thrive in open sections of the same stream that have much greater light intensity. Specific adaptations are required to contend with reactive oxygen species accumulation and not overwhelm photoprotective proteins during transitions from low light to high light (Vass 2012). Therefore, potential future research might examine the transcriptomic abundance of genes encoding the four subunits of PS I and associated photoprotective proteins in combination with ultrastructural studies of the plastid to gain a greater comprehension of this range of tolerance.

The *pet*F data showed significant down‐regulation in shade‐acclimated thalli exposed to high light. Likewise, Nan et al. (2018) reported an up‐regulation of *pet*B, *pet*C, and *pet*H in *Sheathia arcuata* thalli in low‐light relative to high‐light intensities (272 vs. 1,462 μmol photons · m^−2^ · s^−1^, respectively). Although they have different functional roles within the photosynthetic apparatus, these genes are all involved with electron transfer, which is a critical component to proper regulation of photosynthesis (Foyer et al. 2012). The PetF ferredoxin is involved with photophosphorylation in PS I and the photoreduction of NADP+ (Richard et al. 2000, Jacobs et al. 2008), suggesting a close functional relationship between PS I and *pet*F. Similarly, ferredoxins in the green alga, *Chlamydomonas reinhardtii*, have also been shown to play an important role as electron acceptors during anaerobic metabolism with transcription likely anaerobically induced (Happe and Naber 1993, Jacobs et al. 2008). A dual function of *pet*F in red algal plastids may also be present with potential evidence in data collected for the highly reduced plastid genome of a red algal parasite. *Choreocolax polysiphoniae* has lost all genes associated with photosynthesis except *pet*F in its plastid (Salomaki et al. 2015). The retention of *pet*F in all red plastid genomes may indicate other roles of ferredoxin in the organelle. Importantly, regardless of its potential multiplicative role in the plastid, the current study shows *pet*F transcription is significantly affected by light quantity. Investigation of *pet*F transcriptional regulation in other environmental conditions such as during metabolic stress, and in non‐photosynthetic species that still bear plastids, may provide insights for PetF ferredoxin function and regulation in the plastid.

In red algae, the structure of PS II differs from green algae and plants because phycobilisomes form the main light‐harvesting antenna (Gantt 1990). Photosystem II units in green plastids can only absorb short wavelengths of light, whereas the structure of red algal phycobilisomes allows for shorter (phycocyanin) and longer (phycoerythrin) wavelength absorption (Gantt 1990). These differences may influence differential strategies between organisms bearing green and red plastids, such that changes in the ratio of phycocyanin and phycoerythrin may provide the red algal PS II with a unique adaptation to changes in light quantity and quality. A previous ecological study suggested changes in the ratio of phycoerythrin to phycocyanin as a mechanism for photoacclimation to highly variable light intensities, which would likely impact PS II (Kaczmarczyk and Sheath 1991). Although the first transcriptome analysis of a freshwater red alga (*Sheathia arcuata*) at variable irradiances indicated up‐regulation in the transcripts of *cpc* and *cpe* genes at low light, the expression was not significantly different (Nan et al. 2018). Likewise, in this study, we have determined interesting patterns of differential expression in two subunit genes from these families (*cpc*A, *cpe*A) that did not differ significantly. The inverse regulatory pattern observed between these two genes at high light indicates a change in the ratio of the proteins that form light‐harvesting rods of the phycobilisome on PS II. These data corroborate a decrease in total pigment content documented in other studies (Ritz et al. 2000, Aigner et al. 2017), and in the observed disappearance of phycoerythrin hexamers in the light‐harvesting rods of the phycobilisome in *Rhodella violacea* during exposure to higher irradiances (Ritz et al. 2000).

The minor differences in transcription observed in PS II genes relative to PS I suggest that PS I must undergo greater modifications based on changes in light intensity and that adaptations related to photoacclimation in PS II may not be controlled at the transcriptional level. Increasing the number of replicates and measuring transcriptional changes over a shorter temporal period, combined with the incorporation of techniques for photopigment isolation, may increase our understanding of photosystem and phycobilisome modifications that allow freshwater red algal species to occupy highly variable light environments.

### Plastid genome

Red algal and glaucophyte plastids are unique in that light harvesting is accomplished with only one chlorophyll type (chl*. a*; Gantt 1990, Busch et al. 2010). Moreover, the genomes of red algal plastids are among the largest sequenced and are evolutionarily stable, indicating they are most likely the closest extant representatives of the ancestral cyanobacterium that gave rise to the organelle (Janouškovec et al. 2013, Muñoz‐Gómez et al. 2017). In this study, sequencing and assembly of the Lympha *mucosa* plastid genome was a useful tool to design highly specific primers for targeted light‐harvesting genes. In addition, these data also contribute to the increasing data set of red algal plastid genomes that is needed to complete comparative studies of plastid evolution and its implications for red algal physiology and ecology.

The one unique gene in Lympha *mucosa* relative to other freshwater red algal plastids*, ycf37*, has a putative function in red algal plastids and appears to be lost in different taxa across the Florideophyceae (e.g., Salomaki et al. 2015, Verbruggen and Costa 2015, Costa et al. 2016, Lee et al. 2016). However, Wilde et al. (2001) characterized the cyanobacterial *ycf*37 as being involved with PS I assembly and stability, with its inactivation causing a lower ratio of PS I to PS II, and a higher ratio of phycocyanin to chlorophyll. The expression of *ycf*37 in the *L. mucosa* plastid may play a role in the modifications of PS I to changes in irradiance; however, the amino acid translation of the *L. mucosa ycf*37 is highly divergent to its closest relatives that have not lost it from the plastid genome (18–27% similarity). Therefore, *ycf*37 may represent a pseudogene with no putative function and a transcriptional investigation would be required to test this hypothesis.

Highly conserved red algal plastid genomes suggest that differences in photoadaptive strategies between genera, and even species, may be regulated at the transcriptional level. Two of the four known transcription factors encoded in other red algal plastids (*omp*R [*ycf*27]*, ycf*29*;* Minoda et al. 2005) are encoded in the Lympha *mucosa* plastid genome and in other sequenced plastid genomes of Nemaliophycidae (Costa et al. 2016, Lee et al. 2016, Paiano et al. 2017). Complete protein synthesis within the plastid makes these transcription factors ideal for activating rapid transcriptional responses of plastid‐encoded genes, and this may contribute to the greater degree of transcriptional regulation in red algal plastids relative to green algal and land plant chloroplasts, which have lost these response regulatory proteins (Minoda et al. 2005, Riediger et al. 2018). The results from the relative expression study presented here, which were made possible by utilizing the plastid genome as a tool, show an active role of transcriptional changes for the regulation of several light‐harvesting genes after exposure to low light and high light in freshwater red algae.

### Ecophysiological implications

Previously, distinct photoacclimation strategies have been identified for freshwater red algal species. Photosynthetic responses based on PI curves ranging from 20 to 427 μmol photons · m^−2^ · s^−1^ have identified at least two strategy types that are correlated with gross morphology; species or life histories with a tuft morphology became light saturated more quickly and had lower photosynthetic efficiency than the mucilaginous, filamentous morphology (Necchi and Zucchi 2001, Drerup et al. 2015). *Lympha mucosa* is filamentous and has a high Pmax and photosynthetic efficiency (as *Batrachospermum* sp.; Drerup et al. 2015), and its distribution and abundance in different light environments within the same stream indicates it is highly successful in a wide range of light intensities. Therefore, *L. mucosa* is not only adapted for shaded environments but also be adapted to light environments. These measurable differences in photoacclimation may play an important role in shaping the biogeographic and dispersal patterns of freshwater red algal species, with some species able to successfully exploit a range of habitats with variable light regimes, such as in temperate forests.

The ecophysiological evidence for photoacclimation in freshwater red algae includes adjustments in light‐harvesting structures and total pigment content, but there are little genetic, biochemical, or physiological data available to determine underlying cellular mechanisms for this strategy. In this study, the changes in the relative transcript abundance in *psa*A*, pet*F*,* and *cpe*A suggest a level of transcriptional regulation of photoacclimation in freshwater red algae. Given that chloroplast genes are often excluded from relative expression analyses due to poly‐A selection, this serves as an important note that many regulatory activities occur in the chloroplast that need to be understood. However, there are hundreds of genes involved with light harvesting that are encoded in the plastid and nuclear genomes of photosynthetic eukaryotes, including the Rhodophyta, and some of these genes are also significantly differentially regulated at varied irradiances (Engelken et al. 2010, Grouneva et al. 2013, Nan et al. 2018). Furthermore, an examination of post‐transcriptional modifications is needed to provide the necessary evidence for these proposed mechanisms of achieving photoacclimation in these freshwater inhabiting members of the Rhodophyta.

The authors would like to thank the Ohio University Genomics Facility for generating sequencing data and providing equipment for RT‐qPCR, Dr. Sarah Wyatt and Colin Kruse for help with RNA extractions and troubleshooting, and Dr. Erin Murphy for providing equipment for additional RT‐qPCR work. This research was supported through Ohio University research funds to MLV and Ohio University Graduate Student Senate Original Work Grants to JRE. The authors declare no conflicts of interest.

## Supporting information


**Figure S1**. Experimental design for the light treatment of Lympha *mucosa* thalli collected from different site types of the Kinniconick River, KY. Twelve samples were collected from Shade (SH) and Sun (SA) locations and equally split into Low (LL) and High (HL) light conditions. These thalli were cultured at the specific conditions for 72 h before being culled for RNA extraction and transcript quantification of the target genes. Comparisons were performed for each acclimation type at high and low light.

**Figure S2**. The ≥189,825 bp Lympha *mucosa* plastid genome. The genome contains 239 genes with an average GC content of 28.4% The genome encodes 19 protein‐coding genes (function indicated by color), 6 rRNA genes that form two inverted ribosomal operons (red), and 33 tRNA genes encoding anti‐codons for all 20 amino acids (not shown). Two highly conserved group II introns (*chl*B, tRNA‐Met) were detected.

**Table S1**. RT‐qPCR optimization data for each gene analyzed in this study. All genes had successful optimization except *apc*A, which could not have ~100% PCR efficiency obtained.

**Table S2**. Raw Cq data for all gene targets analyzed with RT‐qPCR. Columns contain, from left to right: Well (location on 96‐well PCR plate), Fluor (fluorescent dye used), Content (sample replicate number), Target (gene target), Sample, Threshold Cycle (raw Cq), C(t) Mean (average of technical replicates for a sample), C(t) Std. Dev., Instrument.

**Table S3**. Output data results of the statistical analysis using a two‐way ANOVA and a post hoc Tukey HSD. All data were assessed for normality using a Shapiro‐Wilks test. Categories and values deemed significant are in bold. HL = High Light, LL = Low Light, SA =  Sun‐acclimated, SH – Shade‐acclimated. See Figure S1 for abbreviation details.
