# Supplementary material for: Relative expression analysis of light‐harvesting genes in the freshwater alga Lympha mucosa (Batrachospermales, Rhodophyta)
Source: J Phycol. 2020 Feb 10;56(2):540–8. doi: 10.1111/jpy.12967 (PMC9290634; doi:10.1111/jpy.12967)
Supplement: Supplementary file 1 — Figure S1. Experimental design for the light treatment of Lympha mucosa thalli collected from different site types of the Kinniconick River, KY. Twelve samples were collected from Shade (SH) and Sun (SA) locations and equally split into Low (LL) and High (HL) light conditions. These thalli were cultured at the specific conditions for 72 h before being culled for RNA extraction and transcript quantification of the target genes. Comparisons were performed for each acclimation type at high and low light. [file JPY-56-540-s004.pdf]

Experimental Design

In Stream Collection

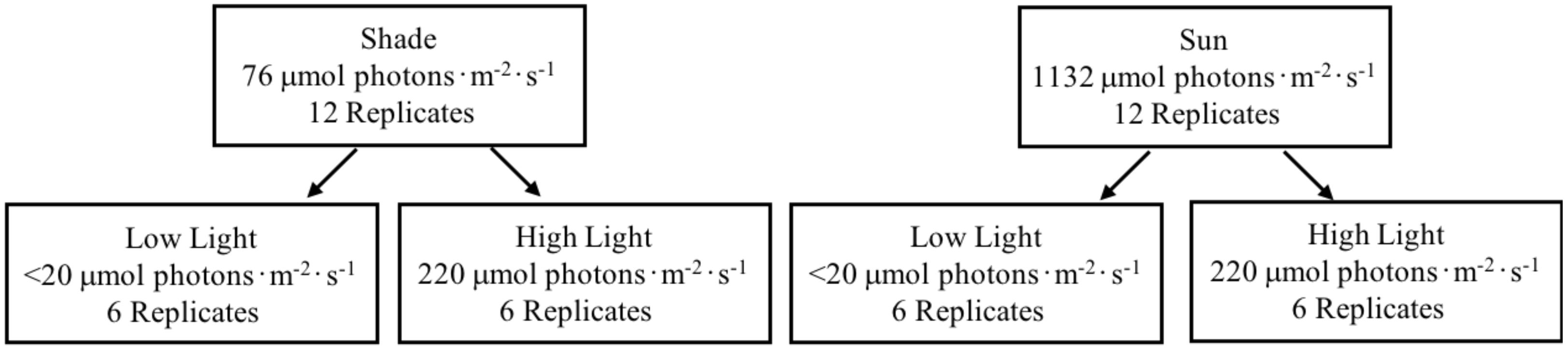

Growth Chamber – 72 h

Figure S1. Experimental design for the light treatment of *L. mucosa* thalli collected from different site types of the Kinniconick River, KY. Twelve samples were collected from Shade (SH) and Sun (SA) locations and equally split into Low (LL) and High (HL) light conditions. These thalli were cultured at the specific conditions for 72 hours before being culled for RNA extraction and transcript quantification of the target genes. Comparisons were performed for each acclimation type at high and low light.
