# Supplementary material for: Relative expression analysis of light‐harvesting genes in the freshwater alga Lympha mucosa (Batrachospermales, Rhodophyta)
Source: J Phycol. 2020 Feb 10;56(2):540–8. doi: 10.1111/jpy.12967 (PMC9290634; doi:10.1111/jpy.12967)
Supplement: Supplementary file 2 — Figure S2. The ≥189,825 bp Lympha mucosa plastid genome. The genome contains 239 genes with an average GC content of 28.4% The genome encodes 19 protein‐coding genes (function indicated by color), 6 rRNA genes that form two inverted ribosomal operons (red), and 33 tRNA genes encoding anti‐codons for all 20 amino acids (not shown). Two highly conserved group II introns (chlB, tRNA‐Met) were detected. [file JPY-56-540-s001.docx]

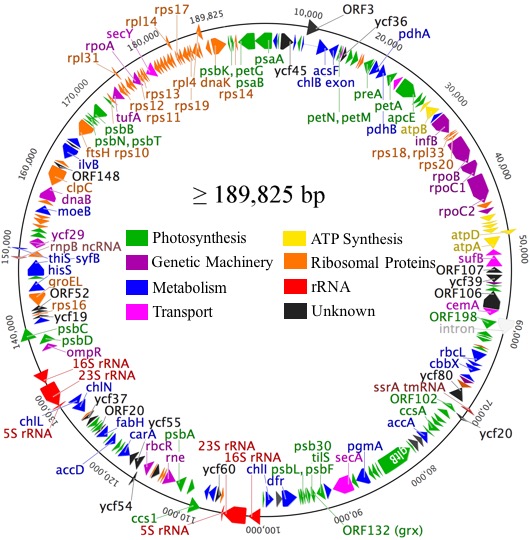


Fig. S2. The ≥ 189,825 bp *Lympha mucosa* plastid genome. The genome contains 239 genes with an average GC content of 28.4%. The genome encodes 198 protein-coding genes (function indicated by color), 6 rRNA genes that form two inverted ribosomal operons (red), and 33 tRNA genes encoding anti-codons for all 20 amino acids (not shown). Two highly conserved group II introns (*chlB,* tRNA-Met) were detected.
