# Supplementary material for: Relative expression analysis of light‐harvesting genes in the freshwater alga Lympha mucosa (Batrachospermales, Rhodophyta)
Source: J Phycol. 2020 Feb 10;56(2):540–8. doi: 10.1111/jpy.12967 (PMC9290634; doi:10.1111/jpy.12967)
Supplement: Supplementary file 3 — Table S1. RT‐qPCR optimization data for each gene analyzed in this study. All genes had successful optimization except apcA, which could not have ~100% PCR efficiency obtained. [file JPY-56-540-s002.docx]

| Gene (Protein) | PCR efficiency (%) | R^2^ | C_t_ range |
| --- | --- | --- | --- |
| *apcA* (Allophycocyanin alpha chain) | 143.1 | 0.999 | 24.07-29.50 |
| *HV60* (Low molecular mass early light-inducible protein HV60) | 82.7 | 0.999 | 23.38-34.14 |
| *cpcA* (Phycocyanin) | 104.7 | 0.995 | 25.50-30.11 |
| *cpeA* (Phycoerythrin) | 87.1 | 0.999 | 17.32-30.04 |
| *petF* (PetF ferredoxin) | 106.6 | 0.993 | 20.97-30.04 |
| *psaA* (Photosystem I alpha subunit) | 80.0 | 0.999 | 18.36-32.07 |
| *psbA* (Photosystem II protein D1) | 91.1 | 0.999 | 18.22-30.73 |
| *eRF3* (Ethylene-responsive transcription factor 3) | 90.4 | 0.995 | 29.98-37.41 |
| *rps3* (Ribosomal Protein S3) | 100.7 | 0.993 | 26.54-33.67 |

Table S1. RT-qPCR optimization data for each gene analyzed in this study. All genes had successful optimization except *apc*A, which could not have ~100% PCR efficiency obtained.
