# Supplementary material for: Relative expression analysis of light‐harvesting genes in the freshwater alga Lympha mucosa (Batrachospermales, Rhodophyta)
Source: J Phycol. 2020 Feb 10;56(2):540–8. doi: 10.1111/jpy.12967 (PMC9290634; doi:10.1111/jpy.12967)
Supplement: Supplementary file 5 — Table S3. Output data results of the statistical analysis using a two‐way ANOVA and a post hoc Tukey HSD. All data were assessed for normality using a Shapiro‐Wilks test. Categories and values deemed significant are in bold. HL = High Light, LL = Low Light, SA = Sun‐acclimated, SH – Shade‐acclimated. See Figure S1 for abbreviation details. [file JPY-56-540-s003.docx]

Table S3. Output data results of the statistical analysis using a two-way ANOVA and a *post hoc* Tukey HSD. All data were assessed for normality using a Shapiro-Wilks test. Categories and values deemed significant are in bold. HL = High Light, LL = Low Light, SA = Sun-acclimated, SH = shade-acclimated. See Figure S1 for abbreviation details.

|  | **Shapiro-Wilks** | | **Two-way ANOVA** | | | | | | **Tukey HSD** | | | | | |
| --- | --- | --- | --- | --- | --- | --- | --- | --- | --- | --- | --- | --- | --- | --- |
| **Gene** | **W** | **p-value** |  | **Df** | **Sum Sq** | **Mean Sq** | **F value** | **P-value** |  | **Diff** | **lwr** | **upr** | **p-value** |  |
| *HV60* | 0.97527 | 0.9575 | HL vs. LL | 1 | 2.4120 | 2.41203 | 4.9092 | 0.05757 | HLSA vs. LLSA | -1.3483333 | -3.1811146 | 0.4844479 | 0.1640566 |  |
|  |  |  | SA vs. SH | 1 | 0.0645 | 0.06453 | 0.1313 | 0.72643 | HLSH vs. LLSH | -0.4450000 | -2.2777812 | 1.3877812 | 0.8625302 |  |
|  |  |  | Interaction between factors | 1 | 0.6120 | 0.61201 | 1.2456 | 0.29679 |  | | | | | |
|  |  |  | Residuals | 8 | 3.9307 | 0.49133 |  | |  |  |  |  |  |  |
| *psa*A | 0.92811 | 0.3605 | **HL vs. LL** | 1 | 50.062 | 50.062 | 78.3599 | **2.093e-05** | **HLSA vs. LLSA** | -2.811667 | -4.9015847 | -0.7217486 | **0.0110747951** |  |
|  |  |  | SA vs. SH | 1 | 0.935 | 0.935 | 1.4639 | 0.2609 | **HLSH vs. LLSH** | -5.358333 | -7.4482514 | -3.2684153 | **0.0001670819** |  |
|  |  |  | **Interaction between factors** | 1 | 4.864 | 4.864 | 7.6137 | **0.0247** |  | | | | | |
|  |  |  | Residuals | 8 | 5.111 | 0.639 |  | |  |  |  |  |  |  |
| *psb*A | 0.96793 | 0.8879 | HL vs. LL | 1 | 3.0100 | 3.0100 | 2.4271 | 0.1579 | HLSA vs. LLSA | -1.668333 | -4.580129 | 1.243462 | 0.324579 |  |
|  |  |  | SA vs. SH | 1 | 2.1252 | 2.1252 | 1.7137 | 0.2269 | HLSH vs. LLSH | -0.335000 | -3.246796 | 2.576796 | 0.9817174 |  |
|  |  |  | Interaction between factors | 1 | 1.3333 | 1.3333 | 1.0751 | 0.3301 |  | | | | | |
|  |  |  | Residuals | 8 | 9.9212 | 1.2402 |  | |  |  |  |  |  |  |
| *pet*F | 0.95906 | 0.7704 | **HL vs. LL** | 1 | 6.1061 | 6.1061 | 22.4451 | **0.001468** | HLSA vs. LLSA | -0.8583333 | -2.22211906 | 0.5054524 | 0.258809985 |  |
|  |  |  | SA vs. SH | 1 | 0.0588 | 0.0588 | 0.2161 | 0.654390 | **HLSH vs. LLSH** | -1.9950000 | -3.35878573 | -0.6312143 | **0.006836197** |  |
|  |  |  | Interaction between factors | 1 | 0.9690 | 0.9690 | 3.5619 | 0.95823 |  | | | | | |
|  |  |  | Residuals | 8 | 2.1764 | 0.2720 |  | |  |  |  |  |  |  |
| *cpc*A | 0.90646 | 0.1922 | HL vs. LL | 1 | 5.2404 | 5.2404 | 3.0203 | 0.1204 | HLSA vs. LLSA | 0.8883333 | -2.55786 | 4.332452 | 0.8408581 |  |
|  |  |  | SA vs. SH | 1 | 0.0520 | 0.0520 | 0.0300 | 0.8668 | HLSH vs. LLSH | 1.7550000 | -1.689119 | 5.199119 | 0.4146510 |  |
|  |  |  | Interaction between factors | 1 | 0.5633 | 0.5633 | 0.3247 | 0.5845 |  | | | | | |
|  |  |  | Residuals | 8 | 13.8803 | 1.7350 |  | |  |  |  |  |  |  |
| *cpe*A | 0.8997 | 0.1572 | **HL vs. LL** | 1 | 10.6597 | 10.6597 | 8.3487 | **0.02022** | HLSA vs. LLSA | -1.63166667 | -4.586185 | 1.3228514 | 0.3529347 |  |
|  |  |  | SA vs. SH | 1 | 0.3571 | 0.3571 | 0.2797 | 0.61128 | HLSH vs. LLSH | -2.13833333 | -5.092851 | 0.8161847 | 0.1728676 |  |
|  |  |  | Interaction between factors | 1 | 0.1925 | 0.1925 | 0.1508 | 0.70791 |  | | | | | |
|  |  |  | Residuals | 8 | 10.2145 | 1.2768 |  | |  |  |  |  |  |  |
